# Health and health-related behaviors in esports players: an international survey of playtime and nationality differences

**DOI:** 10.3389/fspor.2026.1850349

**Published:** 2026-07-08

**Authors:** Oliver Leis, Alexandra Ziegeldorf, Nina Hottenrott, Lara Wunsch, Benjamin T. Sharpe, Michael Geoffrey Trotter, Matthew Watson, Tobias Scholz, Marcelo Moriconi, Despoina Ourda, Leonidas Karaiskos, Vassilis Barkoukis

**Affiliations:** 1 Sport Psychology, Leipzig University, Leipzig, Germany; 2 Institute of Psychology, Business, and Human Sciences, University of Chichester, Chichester, United Kingdom; 3 Department for Health and Sport, Halmstad University, Halmstad, Sweden; 4 Psychology, German Sport University Cologne, Cologne, Germany; 5 Department of Information and Communication Technology, University of Agder, Kristiansand, Norway; 6 Centre for International Studies (CEI-Iscte), ISCTE - Instituto Universitário de Lisboa, Lisboa, Portugal; 7 Department of Physical Education and Sport Science Aristotle University of Thessaloniki, Thessaloniki, Greece; 8 KEA Fair Play Code, Athens, Greece

**Keywords:** competitive gaming, mental health, physical activity, quality of life, sleep

## Abstract

**Introduction:**

Esports participation is expanding rapidly, yet evidence on players' health and health-related behaviors remains limited and inconsistent. This study examined health indicators and health-related behaviors in esports players and explored differences by weekly playtime and nationality.

**Methods:**

A cross-sectional online survey was completed by 413 esports players from 48 nationalities. Measures included body mass index (BMI), physical activity, smoking, alcohol use, sleep behavior, depressive symptoms, health-related quality of life (HRQoL), and perceived social support.

**Results:**

Overall, the sample showed a heterogeneous but not uniformly adverse health profile. Mean BMI was within the normal-weight range, smoking and alcohol consumption were generally low, depressive symptoms were predominantly minimal to mild, and average sleep duration was approximately 8.2 h per night. However, 38.3% of participants were categorized as insufficiently physically active. Weekly playtime was largely unrelated to health indicators. The only significant difference was smoking frequency, with players reporting more than 10 h of weekly playtime smoking slightly more often than those playing 0–10 h. In contrast, nationality was associated with differences in BMI, smoking frequency, drinking frequency, physical activity, and selected HRQoL domains, while no nationality differences were observed for sleep parameters, depressive symptoms, or perceived social support.

**Discussion:**

These findings challenge simplified assumptions that esports players exhibit uniformly poor health and suggest that health-related differences may be better explained by subgroup characteristics and sociocultural context than by gaming volume alone. Future longitudinal and intervention-based research is needed to better understand and promote healthy participation in esports.

## Introduction

Esports continues to grow culturally, economically, and as a professional pursuit [e.g. (1),]. According to Pedraza-Ramirez et al. [(2), p. 4], esports can be defined as “the casual or organized competitive activity of playing specific video games that provide professional and/or personal development to the player”. Given the rapid expansion of esports, understanding how a digital and competitive lifestyle relates to players’ health and health-related behaviors has become increasingly relevant. Historically, esports players have been associated with health-compromising behaviors and an increased risk for poor health. In recent years, research has begun to examine their health and health-related behaviors [e.g. (3, 4),], but significant questions remain. For example, a national study in Germany (3) reported that most players described their health as good to excellent, exercised more than 2.5 h per week, slept approximately seven hours, and played an average of 25 h weekly. Similarly, an international survey by Trotter et al. (4) found that the majority of players were non-smokers (92%) and low consumers of alcohol (65%), although many failed to meet global physical activity recommendations. Whereas Trotter et al. (4) found that the top 10% of players were both the most physically active and the most engaged in gaming, DiFrancisco-Donoghue et al. (5) reported that collegiate competitive esports players were generally less active than their non-competitive peers.

Other areas such as injuries, sleep quality, dietary habits, and doping practices also remain under-researched in esports. Broader reviews further emphasize multiple adverse outcomes [e.g. (6, 7),]. Shulze et al. (6) noted that, despite the growing popularity and professionalization of esports, research on biopsychosocial risks remains limited, highlighting challenges such as poor nutrition, injury, cognitive fatigue, stress, gaming-related disorder, and mental health difficulties, while common assumptions about sedentary behavior or high BMI may be overstated. Similarly, Yin et al. (7) identified issues such as stress, sleep disturbances, eye fatigue, musculoskeletal pain, overuse injuries, metabolic disorders, weight gain, and behavioral issues (e.g., aggression, gaming addiction). For instance, surveys of college students who played esports intensively (5.5–10 h daily) reported eye strain as well as pain in the neck, back, wrist, and hand. Extending this, Poulus et al. (8) identified distinct burnout profiles among esports players, with over one-third classified as high burnout risk. Higher mental toughness and resilience were associated with lower burnout, indicating potential protective factors. More broadly, Poulus et al. (9) demonstrate that mental health in esports is dynamic, with well-being indicators (e.g., intrinsic motivation, self-efficacy) co-occurring with symptoms such as anxiety and gaming addiction. Prevalence estimates vary widely (e.g., anxiety: 38%–82%; depressive symptoms: 25%–37%) and depend on factors such as experience, coping, and competitive context, while research remains predominantly cross-sectional with limited longitudinal and intervention evidence. Similarly, Chan et al. (10) found that higher gaming engagement is associated with poorer diet, reduced physical activity, and suboptimal sleep. Despite these risks, esports participation may also foster positive psychosocial outcomes, including motivation, social relationships, teamwork, and skill development [e.g. (11),]. These mixed findings suggest that esports participation may involve both potential risks and benefits, underscoring the importance of better understanding players’ health behaviors. Major health organizations, including the World Health Organization, advocate for reducing sedentary behavior and promoting physical activity. However, current public health strategies rarely account for emerging youth cultures such as esports. Most guidance focuses broadly on screen time, overlooking the specific demands and routines of competitive gaming [e.g. (12),]. As a result, esports players may be overlooked in health-promotion efforts, highlighting the need for evidence-based guidelines, support systems, and targeted interventions that reflect the realities of esports participation. Consistent with the World Health Organization's understanding of health as encompassing physical, mental, and social well-being, esports players’ health should be considered across multiple domains rather than through isolated indicators alone.

Taken together, existing survey-based research suggests that esports players’ health cannot be characterized in uniformly negative terms. However, much of the current evidence remains fragmented, with studies often focusing on selected health domains, specific national or competitive contexts, or isolated behavioral indicators. Less is known about how multiple physical, behavioral, psychological, and social health indicators present within an international sample, and whether differences in these indicators are more strongly associated with gaming-related involvement, such as weekly playtime, or broader contextual characteristics, such as nationality. Therefore, the present study aimed to provide a multi-dimensional assessment of health and health-related behaviors among esports players. Building on prior research [e.g. (3, 4),], we examined not only demographic and esports-related characteristics but also a broad range of physical and mental health indicators, including BMI, physical activity, smoking, alcohol use, sleep quality, depressive symptoms, health-related quality of life (HRQoL), and perceived social support [e.g. (7),]. This approach allows for a more nuanced understanding of players’ health, moving beyond stereotypes of esports players as uniformly sedentary or unhealthy. Specifically, we sought to address the following research questions: i) What patterns of health indicators and health-related behaviors can be observed among esports players? ii) How do these patterns differ as a function of playtime and other gaming-related characteristics (e.g., game titles)? iii) To what extent do health indicators and health-related behaviors vary across countries? By addressing these questions, the study aimed to inform future research directions and to guide practical interventions, health promotion strategies, and the development of esports-specific guidelines or codes of conduct. Ultimately, this approach aimed at supporting healthier and more sustainable participation in esports.

## Materials and methods

### Design

This study employed a cross-sectional survey design to examine health and health-related behaviors among esports players. The survey was developed using established and validated measures from prior esports and health research. All study procedures were approved by the lead author's university research ethics committee (2024.01.28_eb_vv_25).

### Procedure

Participants were recruited using a multi-channel approach. Recruitment efforts included posts on social media platforms associated with the research project and individual team members (e.g., X, LinkedIn). For instance, three major posts from the project's official X account reached an average of 2,933.3 views and received an average of 12.3 reposts (*SD* = 2.5). In addition to social media outreach, the survey was distributed via email through the research team's professional and academic networks (e.g., students, colleagues), and shared on Discord servers frequented by esports players and organizations. These included local, national (e.g., German Esports Federation), and international esports bodies such as the Esports Research Network. The Global Esports Federation also circulated the survey via email to its members. Moreover, the study was promoted on the [blinded podcast name], which reaches an audience of [blinded metric].

Participants accessed the online survey, provided informed consent, and took an average of 9 min and 35 s to complete it. Data collection occurred between February and July 2025, with an average of 148 individuals accessing the survey each week. A total of 228 individuals discontinued the survey after providing consent on the demographic data page and were therefore excluded from further analysis. Of the participants who proceeded, 420 completed the questionnaire. However, seven responses were excluded as five participants reported not playing esports, and two were removed due to failing plausibility checks. This resulted in a final sample of 413 valid cases.

### Instruments

The survey was informed by previous research [e.g. (3, 4),] and developed through a collective process within the research team. For instance, two authors first discussed the use and selection of specific questionnaires. Subsequent discussion with the entire research team and additional experts providing feedback led to the inclusion of additional questions on participants demographics and sleep behaviors.

#### Demographic data

The survey collected demographic data, including age, gender, body mass index (kg/m^2^), nationality, country of residence, and employment status (i.e., full-time, part-time, self-employed, unemployed, student, retired, prefer not to say), with multiple responses permitted (e.g., combining part-time employment with student status).

#### Esports-related data

The survey assessed esports-related background information, including the specific esports title, years of experience in that title, average weekly playing time (in hours), and current in-game rank. In terms of competition level, the classification by Poulus et al. (13) as: non-competitive (do not compete, beside ranked games), regional (high-school, university, community leagues), second-tier national competition (not the premier national competition), top-tier national competition (highest national standard), and international competition (highest standard).

#### Health indicators and health-related behaviors

**Body Mass Index.** The BMI was calculated from participants’ height and weight using the kg/m^2^ formula. In accordance with the WHO Consultation on Obesity and World Health Organization (14), participants’ BMIs were categorized as Underweight (<18.5), Normal weight (18.5–24.99), Overweight (25–29.99), and Obesity (>30).

**Smoking and Drinking Behavior.** The quantity of smoking cigarettes and drinking alcohol was assessed by recording the number of days including this behavior, ranging from zero days to seven days a week. The quantity, such as the number of drinks, was not collected. Participants were categorized with respect to smoking behavior as non-smokers (0 days), occasional smokers (1–6 days), and daily smokers (7 days per week). Based on the classification by Richter et al. (15), participants’ alcohol consumption was categorized into no risk (0 days), lower risk (1–2 days), moderate risk (3–4 days), and higher risk (more than 4 days per week).

**Physical Activity.** Physical activity was measured using the Godin Leisure-Time questionnaire, asking players to report how many times on average they engaged in certain types of exercise during a typical 7-day period [i.e., “During a typical 7-Day period (a week), how many times on average do you do the following kinds of exercise for more than 15 min?”]. Players were asked to report the average number regarding strenuous, moderate, and mild/light exercise. As seen in the Supplementary Material, brief explanations were provided for each of the three aspects (strenuous exercise: running, football, judo; moderate exercise: fast walking, volley, easy swimming; mild/light exercise: yoga, easy walking). Total leisure-time activity scores were calculated as follows: (9 × number of strenuous exercise sessions per week) + (5 × number of moderate exercise sessions per week) + (3 × number of mild/light exercise sessions per week) and categorizes according to the original Godin criteria as “Insufficiently Active/Sedentary” (<14), “Moderately Active” (14–23), or “Active” (≥24) (16).

**Health-Related Quality of Life.** To assess HRQoL, 12 items based on the SF-36 questionnaire were utilized, covering both physical and mental health domains. The items were aggregated into seven subscales, including Physical Functioning, Role Limitations due to Physical Health (hereafter referred to as Role-Physical), Bodily Pain, General Health, Vitality, Social Functioning, and Mental Health, following the guidelines by Ware et al. (17). Subscale scores were transformed to a 0–100 scale, with higher scores indicating better health-related quality of life. A detailed overview of the subscales, including all corresponding items and response scales, is provided in the Supplementary Materials.

**Depressive Symptoms.** Depressive symptoms were assessed using the Patient Health Questionnaire (PHQ-9) (18), by asking participants how often they were bothered by problems such as little interest or pleasure in doing things, feeling tired, or having trouble concentrating over the past two weeks. The questionnaire consists of nine items, each rated on a 4-point Likert scale from 0 (“not at all”) to 3 (“nearly every day”), yielding a total score ranging from 0 to 27. PHQ-9 total scores were categorized according to established cut-offs as follows: 0–4 = minimal or none, 5–9 = mild, 10–14 = moderate, 15–19 = moderately severe, and 20–27 = severe depression.

**Sleep Behavior.** To assess players’ sleep behavior, items of the Pittsburgh Sleep Quality Index (19) was used to assess the sleep quality (“During the past month, how would you rate your sleep quality overall?”), on a 4-point-Likert scale from very good to very bad. In addition, participants were asked to report the time they usually went to bed (“During the past month, what time have you usually gone to bed at night?”), time taken to fall asleep [“During the past month, how long (in minutes) has it usually takes you to fall asleep each night?”], wake-up time (“During the past month, what time have you usually gotten up in the morning?”), and hours of actual sleep (“During the past month, how many hours of actual sleep did you get at night?”).

**Perceived Social Support.** Perceived social support was assessed using the Multidimensional Scale of Perceived Social Support [MSPSS (20)], a 12-item questionnaire designed to evaluate support from three sources: family, friends, and significant others. Each subscale includes four items. Respondents rate statements such as “My friends really try to help me” on a 7-point Likert scale ranging from 1 (“very strongly disagree”) to 7 (“very strongly agree”). Mean scores are calculated for each subscale, and an overall support score is obtained by averaging the responses across all 12 items. Thus, total and subscale scores range from 1 to 7, with higher scores indicating greater perceived social support.

### Data preparation

Data were first extracted into an Excel file and subsequently transferred into an SPSS dataset for further processing. The dataset was then checked for eligibility and plausibility. Missing data were imputed following recommendations for multiple data imputation (21). After applying Little's MCAR test and visually inspecting the pattern of missingness (22), the expectation maximization (EM) algorithm was used to estimate missing values based on the existing valid cases. To evaluate the adequacy of the imputation, the imputed values were analyzed using t tests, which indicated no substantial differences compared to the original data.

### Data analysis

Statistical analyses were conducted on the imputed data set using IBM SPSS Statistics v. 29.0.1.1. The sample characteristics, esports-related data, and health indicators were summarized using descriptive statistics, reported as *M* (*SD*) for continuous measures and *n* (%) for categorical variables. To investigate group differences in health indicators based on weekly playtime, independent-samples *t* tests with bootstrapping (1,000 samples) were conducted. For the 4-point Likert scale used to assess sleep quality, rank-based differences between the two gaming groups were analyzed using a Mann–Whitney *U* test to account for its ordinal nature.

The association between weekly playing time (0–10 h vs. more than 10 h) and the categorized variables of BMI, smoking frequency, drinking frequency, Godin leisure-time activity score, and PHQ-9 total score was examined using the Pearson-chi-square test. All expected cell frequencies were above 5, except for drinking frequency (25% of cells < 5). Consequently, the Fisher-Freeman-Halton exact test was applied for drinking frequency. Effect size was measured using Cramer's V and interpreted according to Cohen (23): V = .10 represents a small effect, V = .30 a medium effect, and V > .50 a large effect.

To compare players from different countries regarding health indicators and health-related behaviors (all treated as continuous variables), we conducted one-way analyses of variance using Welch's ANOVA. This approach was chosen to account for unequal group sizes and potential violations of the homogeneity of variance assumption and was applied consistently across all comparisons. Games–Howell *post hoc* tests were performed for pairwise comparisons, as they are robust to heteroscedasticity and unequal sample sizes. Effect sizes (Cohen's *d* for *t* tests; *η*^2^ for ANOVA) were reported where appropriate. Statistical significance was set at *p* < .05.

To enhance the robustness of the cross-country comparisons, a conservative inclusion criterion based on sample size was applied. Only countries with a minimum sample size of *N* > 30 were retained for analysis. This threshold was chosen because the robustness of the ANOVA *F* test to violations of the homogeneity of variance assumption decreases under conditions of substantially unequal group sizes (24). Excluding smaller groups (*n* ≤ 30) reduced the risk of inflated Type I error rates and ensured more stable estimates of group means and variances.

After applying this criterion, four countries were included in the analysis: United States (*n* = 41), Germany (*n* = 89), Greece (*n* = 91), and Portugal (*n* = 90), alongside an ‘Others’ group (*n* = 102) comprising participants from various other countries. For sleep quality, the Kruskal–Wallis test was employed due to the ordinal nature of the 4-point Likert scale.

## Results

### Descriptive results

A total of 413 participants (83.5% male) took part in the study, with a mean age of 24.03 years (*SD* = 7.34; range 11-59). Participants represented 48 nationalities across six inhabited continents. Greece (22.0%), Portugal (21.8%), Germany (21.5%), and the United States (9.9%) accounted for the largest proportions of the sample.

Regarding employment status, the largest group consisted of students (43.6%), followed by full-time employees (26.9%). Additional categories included part-time employees (8.5%), self-employed individuals (6.8%), students working part-time (5.8%), and unemployed individuals (3.9%). The remaining participants (4.5%) were classified as others, reflecting less frequent employment situations.

Participants reported playing a variety of esports titles. The most frequently mentioned game was League of Legends (30.0%), followed by Fortnite (19.4%), Counter-Strike (10.2%), Valorant (9.2%), and Rocket League (5.3%). The remaining participants (19.6%) reported other games, predominantly EA Sports FC, Overwatch, and Call of Duty.

On average, participants reported 7.60 years of esports experience (*SD* = 5.30) and currently spent 15.65 h per week playing (*SD* = 14.76). Due to a small number of high-frequency players (up to 98 h/week), the median of 10 h per week provides an accurate indicator of central tendency. Overall, 51.8% of participants reported playing 0–10 h per week, whereas 48.2% reported playing more than 10 h per week.

Regarding competition level, the majority identified as non-competitive players (61.5%). Among those competing, 22.8% played at a regional level, 9.7% participated in second-tier national level, 3.1% at the top-tier national level, and 2.9% at the international level (see Table 1).

**Table 1:** General sample description.

Variable	% (n)	M (SD)
Gender		
Female	14.3 (59)	
Male	83.5 (345)	
Diverse	1.7 (7)	
No information	0.5 (2)	
Age		24.03 (7.34)
Nationality		
American	9.9 (41)	
German	21.5 (89)	
Greek	22.0 (91)	
Portuguese	21.8 (90)	
Others (*n* = 44)	24.7 (102)	
Employment status		
Student	43.6 (180)	
Full-time	26.9 (111)	
Part-time	8.5 (35)	
Self-employed	6.8 (28)	
Student & Part-time	5.8 (24)	
Unemployed	3.9 (16)	
Others	4.6 (19)	
Esport games		
League of Legends	30.0 (124)	
Fortnite	19.4 (80)	
Counter-Strike	10.2 (42)	
Valorant	9.2 (38)	
Rocket League	5.3 (22)	
Others	19.6 (81)	
Playing experience (years)		7.60 (5.30)
Playtime (average h/week)		15.65 (14.76)
Playtime (h/week)		
0 to 10	51.8 (214)	
> 10	48.2 (199)	
10.1–20	26.6 (110)	
20.1–30	11.6 (48)	
> 30	9.9 (41)	
Competition level		
Non-competitive	61.5 (254)	
Regional	22.8 (94)	
Second-tier national competition	9.7 (40)	
Top-tier national competition	3.1 (13)	
International competition	2.9 (12)	

*N* = 413. M, mean; SD, standard deviation.

Descriptive statistics on health indicators and health-related behaviors are presented in Table 2. The mean BMI of the sample was 24.81 (*SD* = 5.12). According to BMI classifications, 54.0% were classified as normal weight, 26.2% as overweight, 13.8% as obese, and 6.1% as underweight.

**Table 2:** Descriptive statistics of health indicators and health-related behaviors.

Variable	% (n)	M (SD)
BMI		24.81 (5.12)
BMI categories		
Underweight	6.1 (25)	
Normal weight	54.0 (223)	
Overweight	26.2 (108)	
Obesity	13.8 (57)	
Smoking (days/week)		0.85 (2.08)
Smoking categories		
Non-smokers	82.6 (341)	
Occasional smokers	9.4 (39)	
Daily smokers	8.0 (33)	
Drinking (days/week)		0.69 (1.14)
Drinking categories		
No risk	60.8 (251)	
Lower risk	32.9 (136)	
Moderate risk	4.6 (19)	
Higher risk	1.7 (7)	
Physical activity score		29.08 (37.85)
Physical activity categories		
Active	43.1 (178)	
Moderately active	18.6 (77)	
Insufficiently active	38.3 (158)	
Sleep Behavior		
Sleep quality		2.19 (0.73)
Sleep duration (minutes)/(hours)		492.04 (101.25)/ 8.20 (1.69)
Sleep onset latency (minutes)		23.97 (23.85)
Depressive symptoms score		6.25 (5.80)
Depressive symptoms categories		
Minimal/None	50.6 (209)	
Mild	27.1 (112)	
Moderate	11.6 (48)	
Moderately severe	6.5 (27)	
Severe	4.1 (17)	
HRQoL		
Physical Functioning		76.57 (25.25)
Role-Physical		80.72 (26.22)
Bodily Pain		83.75 (19.44)
General Health		59.35 (24.29)
Vitality		57.51 (18.69)
Social Functioning		29.54 (30.70)
Mental Health		54.42 (23.37)
Perceived social support total score		5.29 (1.34)
perceived social support subscale		
Family		5.17 (1.59)
Friends		5.43 (1.51)
Significant others		5.27 (1.27)

*N* = 413. Smoking and drinking categories were based on days/week, ranging from 0 to 7. Physical activity score was based on minutes of exercise and ranged from 0 to open-ended; categories were insufficiently active (<14), moderately active (14–23), and active (≥24). Sleep quality ranged from 1 to 4, with lower scores indicating better sleep quality. Depressive symptoms ranged from 0 to 27, with higher scores indicating more severe symptoms. HRQoL scores ranged from 0 to 100, with higher scores indicating better health-related quality of life. Perceived social support scores ranged from 1 to 7, with higher scores indicating greater perceived social support. BMI, body mass index; HRQoL, health-related quality of life; *M*, mean; *SD*, standard deviation.

Participants reported an average of 0.85 (*SD* = 2.08) smoking days per week. The majority were non-smokers (82.6%), whereas 9.4% reported occasional and 8.0% daily smoking. Mean drinking frequency was 0.69 days per week (*SD* = 1.14). Most participants were classified as no-risk drinkers (60.8%), followed by lower-risk (32.9%), moderate-risk (4.6%), and higher-risk drinkers (1.7%).

The mean score for physical activity was 29.08 (*SD* = 37.85), with 43.1% of participants classified as active, 18.6% as moderately active, and 38.3% as insufficiently active.

Regarding sleep, the mean subjective sleep quality score was 2.19 (*SD* = 0.73). Average sleep duration was 492.04 min (*SD* = 101.25), equivalent to 8.20 h (*SD* = 1.69), and mean sleep onset latency was 23.97 min (*SD* = 23.85).

The mean score for depressive symptom score was 6.25 (*SD* = 5.80). In terms of severity categories, 50.6% reported minimal or no depressive symptoms, 27.1% mild, 11.6% moderate, 6.5% moderately severe, and 4.1% severe symptoms.

For health-related quality of life, mean scores were 76.57 (*SD* = 25.25) for Physical Functioning, 80.72 (*SD* = 26.22) for Role Physical, 83.75 (*SD* = 19.44) for Bodily Pain, 59.35 (*SD* = 24.29) for General Health, 57.51 (*SD* = 18.69) for Vitality, 29.54 (*SD* = 30.70) for Social Functioning, and 54.42 (*SD* = 23.37) for Mental Health.

The mean total score for perceived social support was 5.29 (*SD* = 1.34). Subscale scores were 5.17 (*SD* = 1.59) for Family, 5.43 (*SD* = 1.51) for Friends, and 5.27 (SD = 1.27) for Significant Others.

### Differences in health indicators and health-related behaviors by playtime amount

Inferential analyses examining differences between weekly playtime groups (0–10 h vs. > 10 h) showed statistically significant differences only for smoking behaviors. An independent samples *t*-test indicated that participants playing more than 10 h per week reported significantly more smoking days per week (*M* = 1.12, *SD* = 2.36) than those playing 0–10 h (*M* = 0.59, *SD* = 1.75), *t*(364.02) = −2.53, *p* = .012. As the assumption of normality was violated, a bootstrap procedure with 1.000 resamples was applied to ensure robustness. The bootstrapped results confirmed the statistical significance [*p* = .019, 95% BCa CI (−0.93, −0.12)]. The effect size was small (*d* = −0.25), indicating slightly higher smoking frequency among participants with more than 10 h of weekly playtime. No other health indicators and health-related behaviors differed significantly between groups (see Table 3).

**Table 3:** Comparison of health variables by weekly esports playtime.

Variable	0–10 h (*n* = 214)	>10 h (*n* = 199)	*t* (*df*)	*p_boot_*	95% CI	*d*
	*M* (*SD*)	*M* (*SD*)				
Health Behavior						
BMI	24.97 (5.26)	24.64 (4.98)	0.65 (411)	.541	[−0.63, 1.22]	.06
Smoking days/week	0.59 (1.75)	1.12 (2.36)	−2.53 (364.02)	.019*	[−0.93, −0.12]	−.25
Drinking days/week	0.53 (0.96)	0.75 (1.31)	−1.08 (360.29)	.278	[−0.36, 0.10]	−.11
Physical activity	30.09 (45.97)	27.99 (26.56)	0.56 (411)	.597	[−4.53, 9.51]	.06
Sleep duration (min)	491.15 (100.06)	493.00 (102.76)	−0.19 (411)	.867	[−21.50, 19.91]	−.02
Sleep onset latency (min)	22.81 (22.66)	25.22 (25.08)	−1.03 (411)	.300	[−7.37, 2.69]	−.10
Depressive symptoms	6.23 (5.80)	6.27 (5.82)	−0.06 (411)	.952	[−1.17, 1.11]	−.01
HRQoL						
Physical Functioning	75.99 (25.32)	77.20 (25.23)	−0.48 (411)	.622	[−5.71, 3.65]	−.05
Role-Physical	80.14 (26.95)	81.34 (25.47)	−0.47 (411)	.638	[−5.89, 3.33]	−.05
Bodily Pain	83.64 (19.93)	83.86 (18.95)	−0.11 (411)	.912	[−3.94, 3.84]	−.01
General Health	60.28 (25.19)	58.35 (23.30)	0.81 (411)	.387	[−2.20, 6.31]	.08
Vitality	56.19 (17.71)	58.92 (19.64)	−1.48 (411)	.130	[−6.38, 1.15]	−.15
Social Functioning	30.49 (31.24)	28.52 (30.15)	0.65 (411)	.482	[−3.64, 7.69]	.06
Mental Health	53.97 (24.14)	54.90 (22.57)	−0.40 (411)	.693	[−5.54, 3.79]	−.04
Perceived social support						
Total score	5.23 (1.39)	5.36 (1.28)	1.01 (411)	.333	[−0.42, 0.16]	−.10
Family	5.18 (1.60)	5.16 (1.59)	0.10 (411)	.918	[−0.31, 0.33]	.01
Friends	5.35 (1.56)	5.52 (1.44)	−1.17 (411)	.232	[−0.46, 0.12]	−.12
Significant others	5.15 (1.83)	5.39 (1.61)	−1.43 (409.67)	.154	[−0.55, 0.07]	−.14

*N* = 413. *p*-values and 95% bias-corrected and accelerated confidence intervals (CI) were derived from 1,000 bootstrap samples. BMI, body mass index; HRQoL, health-related quality of life; d, Cohen's d; h, hours; *M*, mean; *SD*, standard deviation. a t-statistics and degrees of freedom (df) are based on Welch's *t*-test.

*p < .05.

Additional chi-square analyses were conducted to examine associations between weekly playtime (0–10 h vs. > 10 h) and categorized health indicators. No significant association was observed between weekly playtime and physical activity levels (insufficiently active/sedentary, moderately active, active), *χ*^2^(2) = 3.16, *p* = .206, V = .09, indicating a negligible effect.

Similarly, weekly playtime was not significantly associated with drinking frequency categories. Due to violations of the minimum expected cell frequency assumption (25% of cells < 5), significance was determined using the Fisher–Freeman–Halton exact test. The analysis revealed no significant association, *χ*^2^(3) = 3.13, *p* = .379, V = .09 (negligible effect).

Furthermore, no significant association was found between weekly playtime and BMI categories, *χ*^2^(3) = 2.14, *p* = .544, V = .07, indicating a negligible relationship. Likewise, depressive symptom severity categories did not differ significantly between playtime groups, *χ*^2^(4) = 6.29, *p* = .178, although a small effect size was observed (V = .12). Overall, categorical health indicators and behavior did not vary significantly between weekly playtime groups.

Subjective sleep quality was compared between playtime groups using a Mann–Whitney *U* test. As distributional shapes differed between groups, results are reported using mean ranks. No significant difference emerged (*U* = 19,590, *Z* = −1.60, *p* = .110), with a mean rank of 199.04 (0–10 h) and 215.56 (>10 h). The effect size was negligible (*r* = .08), indicating comparable subjective sleep quality across both groups.

### Differences in health indicators and health-related behaviors by nationality

For nationality-based analyses, only countries with more than 30 participants were analyzed separately: United States (*n* = 41), Germany (*n* = 89), Greece (*n* = 91), and Portugal (*n* = 90). Participants from all remaining nationalities were combined into an “Other” category (*n* = 102). Welch ANOVAs revealed significant nationality effects for BMI, smoking frequency, drinking frequency, physical activity, and selected HRQoL subscales (see Table 4). A significant effect emerged for BMI, *F*Welch (4, 164.08) = 3.63, *p* = .007, *η*^2^ = .04. Games–Howell *post hoc* tests indicated that Greek participants (*M* = 25.16, *SD* = 3.50) had a higher BMI than Portuguese participants (*M* = 23.40, *SD* = 3.97, *p* = .016). No further pairwise differences were observed.

**Table 4:** Means, standard deviations, and ANOVA results for health behavior variables by nationality.

Variable	American^1^ (*n* = 41)	German^2^ (*n* = 89)	Greek^3^ (*n* = 91)	Portuguese^4^ (*n* = 90)	Others^5^ (*n* = 102)	*F* (*df1, df2*)	*p*	*η^2^*
	*M* (*SD*)	*M* (*SD*)	*M* (*SD*)	*M* (*SD*)	*M* (*SD*)			
Health Behavior								
BMI	26.99 (8.28)	25.08 (5.11)	25.16 (3.50)^4^	23.40 (3.97)^3^	24.64 (5.32)	3.63 (4, 164.08)	.007*	.04
Smoking days/week	0.41 (1.53)^3^	0.70 (1.83)^3^	1.82 (2.76)^1,2,4,5^	0.66 (1.91)^3^	0.44 (1.66)^3^	4.71 (4, 175.47)	.001*	.07
Drinking days/week	0.12 (0.33)^2,3,4,5^	0.64 (0.94)^1^	1.14 (1.46)^1,5^	0.88 (1.35)^1,5^	0.37 (0.73)^1,3,4^	16.98 (4, 198.95)	<.001*	.09
Physical activity	43.46 (29.87)^2,3^	27.89 (26.28)^1^	18.87 (49.82)^1^	34.24 (40.24)	28.88 (32.37)	3.60 (4, 173.11)	.008*	.03
Sleep duration (min)	511.20 (157.89)	491.47 (75.18)	484.37 (95.91)	486.21 (92.38)	496.83 (105.11)	0.41 (4, 165.26)	.802	.01
Sleep onset latency (min)	26.05 (15.65)	27.31 (27.05)	22.32 (27.65)	20.72 (17.31)	24.55 (24.85)	1.36 (4, 180.197)	.248	.01
Depressive symptoms	7.37 (6.28)	5.54 (5.00)	6.32 (5.30)	5.63 (5.98)	6.90 (6.46)	1.24 (4, 170.36)	.297	.01
HRQoL								
Physical Functioning	64.02 (30.00)^2,4^	80.34 (23.45)^1^	75.82 (25.33)	82.22 (21.45)^1^	74.02 (26.01)	3.99 (4, 168.62)	.004*	.04
Role-Physical	73.48 (28.39)	79.21 (29.55)	79.12 (24.93)	84.03 (24.86)	83.46 (24.13)	1.51 (4, 169.64)	.201	.02
Bodily Pain	78.96 (24.75)	84.13 (19.47)	82.42 (19.09)	87.36 (16.81)	83.33 (19.30)	1.49 (4, 167.70)	.209	.01
General Health	58.54 (28.84)	54.21 (24.57)^3^	64.70 (22.48)^2^	63.75 (22.57)	55.51 (23.90)	3.71 (4, 168.64)	.006*	.03
Vitality	50.61 (18.74)^3,4^	53.23 (20.08)^3^	62.91 (16.92)^1,2^	60.69 (17.73)^1^	56.51 (18.17)	5.43 (4, 171.20)	<.001*	.05
Social Functioning	39.63 (31.11)	28.65 (31.88)	32.97 (29.32)	25.56 (29.98)	26.72 (30.72)	2.00 (4, 171.56)	.097	.02
Mental Health	46.34 (24.72)	57.02 (22.92)	56.87 (22.07)	55.83 (23.42)	51.96 (23.78)	1.99 (4, 170.84)	.099	.02
Perceived social support								
Total score	4.94 (1.70)	5.27 (1.43)	5.49 (1.01)	5.43 (1.30)	5.14 (1.36)	1.79 (4, 166.783)	.134	.02
Family	4.72 (1.91)	5.10 (1.60)	5.37 (1.23)	5.38 (1.70)	5.05 (1.60)	1.60 (4, 167.75)	.176	.02
Friends	5.06 (1.88)	5.48 (1.54)	5.60 (1.26)	5.65 (1.36)	5.20 (1.59)	1.81 (4, 167.83)	.130	.02
Significant others	5.03 (1.95)	5.24 (1.88)	5.51 (1.40)	5.27 (1.76)	5.17 (1.74)	0.87 (4, 168.94)	.481	.01

*N* = 413. F statistics and degrees of freedom (df) are based on Welch's ANOVA. BMI, body mass index; HRQoL, health-related quality of life; *η*^2^ = eta squared; *M* = mean; *SD* = standard deviation.

Superscript numbers indicate a significant difference (*p* < .05) from the group in the corresponding column (1 = American, 2 = German, 3 = Greek, 4 = Portuguese, 5 = Others) according to Games-Howell *post-hoc* comparisons. Physical activity score is based on minutes of exercise from 0 to open-ended. The depressive symptoms score ranges from 0 to 27, with higher scores indicating severe depressive symptoms. HRQoL subscale scores range from 0 to 100, with higher scores indicating better health-related quality of life. Perceived social support total and subscale scores range from 1 to 7, with higher scores indicating greater perceived social support.

*p < .05.

Smoking days per week differed significantly across nationalities, *F*Welch (4, 175.47) = 4.71, *p* = .001, *η*^2^ = .07 (medium effect). Greek participants (*M* = 1.82, *SD* = 2.76) reported significantly more smoking days than Americans (*M* = 0.41, *SD* = 1.53, *p* = .002), Germans (*M* = 0.70, *SD* = 1.83, *p* = .013), Portuguese (*M* = 0.66, *SD* = 1.91, *p* = .010), and participants categorized as “Other” (*M* = 0.44, *SD* = 1.66, *p* < .001). No additional pairwise differences were significant.

Drinking frequency also varied significantly, *F*Welch (4, 198.95) = 16.98, *p* < .001, *η*^2^ = .09. Americans reported fewer drinking days (*M* = 0.12, *SD* = 0.33) than all other nationality groups (all *p* < .05). In addition, participants in the “Other” category (*M* = 0.37, *SD* = 0.73) reported fewer drinking days than Portuguese (*M* = 0.88, *SD* = 1.35, *p* = .016) and Greek participants (*M* = 1.14, *SD* = 1.46, *p* < .001).

Physical activity levels differed significantly across nationalities, *F*Welch (4, 173.11) = 3.60, *p* = .008, *η*^2^ = .03. American participants (*M* = 43.46, *SD* = 29.87) reported higher activity levels than German (*M* = 27.89, *SD* = 26.28, *p* = .042) and Greek participants (*M* = 18.87, *SD* = 49.82, *p* = .006).

For HRQoL, significant nationality effects were observed for General Health, Physical Functioning, and Vitality subcategories. General Health differed across groups, *F*Welch (4, 168.64) = 3.71, *p* = .006, *η*^2^ = .03, with Greek participants (*M* = 64.70, *SD* = 22.48) scoring higher than Germans (*M* = 54.21, *SD* = 24.57, *p* = .027). Physical Functioning also varied significantly, *F*Welch (4, 168.62) = 3.99, *p* = .004, *η*^2^ = .04. Americans (*M* = 64.02, *SD* = 30.00) reported lower functioning than Germans (*M* = 80.34, *SD* = 23.45, *p* = .025) and Portuguese participants (*M* = 82.22, *SD* = 21.45, *p* = .008). Vitality showed a small but significant effect, *F*Welch (4, 171.20) = 5.43, *p* < .001, *η*^2^ = .05. Americans (*M* = 50.61, *SD* = 18.74) reported lower energy levels than Greek (*M* = 62.91, *SD* = 16.92) and Portuguese participants (*M* = 60.69, *SD* = 17.73), and Greeks scored higher than Germans (*M* = 53.23, *SD* = 20.08; all *p* < .05).

No significant nationality effects were observed for sleep duration, sleep onset latency, depressive symptoms, Role-Physical, Bodily Pain, Social Functioning, Mental Health, or any perceived social support subscales (all *p* > .05, *η*^2^ ≤ .02). A Kruskal–Wallis test further indicated no significant differences in subjective sleep quality across nationality groups, *H*(4) = 5.97, *p* = .201, *η^2^* < .01, with comparable median scores across groups.

## Discussion

The present study examined health indicators and health-related behaviors in a large international sample of esports players and explored differences by weekly playtime and nationality. In summary, overall health indicators in this sample were heterogeneous but not uniformly adverse. In addition, nationality appeared more strongly associated with several health behaviors and selected health-related quality of life domains than weekly playtime. Descriptively, the mean BMI corresponded to the WHO-defined normal weight category, although a substantial proportion of participants were classified as overweight or obese. Smoking and alcohol consumption levels were generally low, and depressive symptom severity was predominantly minimal to mild. While more than one-third of participants were categorized as insufficiently active, a comparable proportion met criteria for being physically active. Moreover, sleep duration averaged approximately eight hours per night. Viewed through the World Health Organization's multidimensional understanding of health, these findings suggest that esports players’ health profiles are heterogeneous across physical, behavioral, psychological, and social domains rather than uniformly adverse. However, this interpretation should be considered in light of the sample's weekly playtime distribution, as most participants reported low to moderate weekly playtime. Therefore, the findings may primarily reflect the health profiles of lower-volume esports players rather than those of high-volume, professional, or near-professional players.

The present study adds to existing survey-based research on esports players’ health by providing a multidimensional assessment of physical, behavioral, psychological, and social health indicators in a large international sample. While previous studies have often focused on specific health domains, particular player groups, or single-country samples, the present findings offer a broader overview of health-related patterns across diverse esports players. In particular, the comparison of weekly playtime and nationality contributes to the literature by showing that gaming volume alone may be less informative than broader contextual and individual factors when examining health in esports.

Importantly, weekly playtime was largely unrelated to the assessed health indicators. Except for a small difference in smoking frequency, no significant differences were observed between lower and higher playtime groups across BMI, alcohol use, physical activity, sleep parameters, depressive symptoms, health-related quality of life, or perceived social support. Rather than merely indicating an absence of effects, these findings challenge a commonly implied dose (response assumption in esports) that greater playtime necessarily translates into poorer health outcomes. Instead, weekly playtime alone appears insufficient to account for variability in health outcomes, with individual and contextual factors likely playing a more prominent role.

Our findings therefore do not support the assumption of a uniformly adverse health profile among esports players, nor do they support the notion that higher engagement *per se* constitutes a primary risk factor. Instead, variability across health domains appears more closely linked to individual and contextual characteristics. However, findings should be interpreted within the context of the sample, cross-sectional design, and self-reported data. For instance, Nicholson et al. (25) reported that esports players may overestimate physical activity when assessed using the IPAQ-LF, and broader methodological work has highlighted limitations of questionnaire-based assessments, particularly in younger populations (26). Consequently, the present findings should not be taken as definitive evidence regarding activity sufficiency but instead underscore the need for objective measurement approaches. At the same time, prior research suggests that physical activity may positively relate to esports performance (27), indicating that engagement in exercise could serve performance-related as well as health-related functions.

Sleep duration in the current sample averaged approximately eight hours per night and did not differ by playtime or nationality. This contrasts with research in professional esports contexts reporting delayed sleep timing and disrupted sleep patterns associated with training load and mood (28). Differences in competitive level, training structure, and occupational demands may partly explain this discrepancy, as the current sample consisted predominantly of non-professional players.

Depressive symptom severity was generally low relative to prior reports in competitive esports populations [e.g. (29),]. For instance, Kegelaers et al. (29) observed higher rates of severe depressive symptoms and greater sleep disturbance among competitive players. Although direct comparisons are limited by methodological differences, the present findings further support the interpretation that competitive intensity and performance pressure, rather than gaming volume *per se*, may be more relevant for mental health outcomes in esports contexts.

In contrast to weekly playtime, nationality was associated with differences in several behavioral indicators and selected health-related quality of life domains. These differences likely reflect broader cross-national variation in health behaviors, substance use norms, and sociocultural contexts rather than esports-specific mechanisms. In contrast to weekly playtime, nationality was associated with differences in several behavioral indicators and selected health-related quality of life domains. However, nationality should not be interpreted as an explanatory factor in itself, but rather as a broad proxy for overlapping contextual influences, including sociocultural norms, lifestyle patterns, public health environments, socioeconomic conditions, and differences in esports ecosystems. Thus, the observed nationality-related differences should be understood as indirect indicators of broader contextual variation rather than evidence that nationality itself explains health-related outcomes. For example, smoking and drinking patterns across nationality groups may partly mirror population-level differences. Similarly, BMI differences should be interpreted cautiously, as BMI may not accurately capture health status in esports populations without complementary body composition or cardiometabolic measures (5).

### Limitations

This study has several limitations that should be considered when interpreting the findings. First, the survey was available only in English, which may have restricted participation and introduced comprehension-related bias among non-native English-speaking players. Given the multinational nature of the sample, the cross-cultural comparability of the measures should also be interpreted cautiously. Differences in language proficiency, cultural response styles, and the extent to which scales show equivalent validity across countries may have influenced participants’ responses. Therefore, nationality-related comparisons should be considered exploratory rather than definitive evidence of cross-cultural differences. Second, the recruitment strategy, which relied on social media, professional networks, and esports-related platforms, may have introduced selection bias and overrepresented players who were more engaged, health-conscious, or socially connected. Notable participant dropout during the demographic section may have further affected sample composition. Third, the interpretation of the relatively favorable health indicators should consider the weekly playtime distribution of the sample. More than half of the participants reported playing 0–10 h per week, and 78.4% reported playing 20 h or less per week. Thus, most participants had comparatively low to moderate weekly playtime rather than professional or near-professional training volumes. This may have contributed to the generally favorable health-related profile observed in this study and limits the generalizability of the findings to high-volume or professional esports players. Fourth, several measurement limitations should be acknowledged. The questionnaire was intentionally kept brief to accommodate the time-pressured routines of esports players, which limited the extent to which certain variables could be assessed in detail. In addition, all measures were self-reported, including BMI, physical activity, sleep, smoking, and alcohol use, which may have introduced reporting bias. For example, participants may have overreported socially desirable behaviors such as physical activity or underreported less desirable behaviors such as smoking or alcohol consumption. The use of selected or adapted items from established instruments, rather than full validated scales in all cases, may also limit validity and comparability with previous research. Moreover, smoking and alcohol consumption were assessed only by frequency rather than absolute quantity, and smoking status did not differentiate between never-smokers and former smokers within the “0 days” category. Fifth, the categorization of nationality also presents a limitation. The “Other” category included participants from multiple countries, creating a heterogeneous group that limits the interpretation of country-specific differences. Finally, the cross-sectional design of the study precludes causal inferences regarding the relationship between esports participation and health outcomes.

### Practical implications

The present findings suggest that health risks are not uniform across esports players. In contrast, certain subgroups may be more vulnerable to specific health-related challenges. Consequently, health promotion efforts should move beyond general recommendations and instead focus on targeted interventions tailored to these higher-risk groups. Practical strategies to promote physical activity and healthy routines could include session hygiene practices such as posture checks, micro-breaks, warm-up routines, structured training schedules, and effective time management [e.g. (30),]. Integrating these behaviors into players’ existing training routines may increase feasibility and support long-term adherence. Support structures, including coaches, sport psychologists, and educators, may play an important role in promoting and reinforcing healthy behaviors within esports environments [e.g. (31),]. Providing these stakeholders with targeted education, training, or workshops may strengthen their ability to encourage and monitor positive health practices. In addition, framing health behaviors in performance-oriented terms, such as recovery, consistency, and sustained skill development, may resonate more strongly with players than general health messaging. Finally, esports clubs, universities, and teams may serve as important delivery contexts for health promotion initiatives. By embedding health-supportive practices into daily training environments and organizational cultures, these institutions can help normalize and sustain healthy behaviors among players. Aligning health initiatives with players’ competitive goals may ultimately increase engagement and enhance the effectiveness of such interventions.

### Future research

Given the performance-focused and time-constrained nature of esports, future research would benefit from employing more detailed survey designs and incorporating qualitative approaches to better understand why some players engage in less healthy behaviors. Such approaches may provide deeper insights into contextual and motivational factors influencing health-related behaviors in esports environments [e.g. (9),]. Future studies should also investigate potential differences across specific subgroups. For example, examining gender-related experiences, including those of female players, may provide valuable insights, although recruitment in these groups may be challenging. In addition, comparing players across performance levels, competitive ranks, weekly playtime, and game titles or genres may help clarify whether health indicators differ systematically across different esports contexts. Larger and more balanced samples across countries would further improve the interpretability of geographic comparisons and reduce the need for heterogeneous categories such as “Other.” Given the largely non-significant group differences observed in the present analyses, future research may also benefit from applying person-centered analytical approaches, such as cluster analysis or latent profile analysis. These approaches may help identify distinct health behavior and health profile patterns within esports players, rather than relying solely on predefined grouping variables such as playtime or nationality [e.g. (7),]. Such analyses could provide a more nuanced understanding of how combinations of health behaviors, sleep patterns, and psychological indicators cluster within the esports population. Future research should also explore contextual factors that promote or hinder healthy behaviors in esports, including peer support, time management practices, and the broader social climate within teams or gaming communities. Importantly, research should not be limited to players alone but also include coaches, staff, and other support personnel who may influence players’ routines and health-related behaviors [e.g. (32),]. Finally, intervention-based and longitudinal research designs are needed to better understand how health behaviors evolve over time and how they may be improved. In particular, studies examining interventions targeting physical activity [e.g. (27),], sleep [e.g. (33),], and team-based strategies to support both health and performance [e.g. (34),] may provide valuable guidance for developing effective health promotion strategies in esports.

## Conclusion

In this international sample of esports players, health indicators were heterogeneous but not uniformly adverse. Weekly playtime showed minimal associations with physical or mental health outcomes, whereas nationality was more consistently related to behavioral differences, suggesting that broader sociocultural context may be more relevant than gaming volume alone. Overall, these findings challenge simplified assumptions that greater esports engagement inherently leads to poorer health and highlight the importance of context-sensitive and subgroup-specific approaches in both research and practice. However, longitudinal and objectively measured studies are needed to further clarify the complex relationships between esports participation and health.

## Data Availability

The data that support the findings of this study are available from the corresponding author upon reasonable request.

## References

[1] Jin DY Besombes N. Global history of esports. In: Jenny SE Besombes N Brock T Cote AC Scholz TM, editors. Routledge Handbook of Esports. London/New York: Routledge (2024). p. 19–29.

[2] Pedraza-Ramirez I Musculus L Raab M Laborde S. Setting the scientific stage for esports psychology: a systematic review. Int Rev Sport Exerc Psychol. (2020) 13(1):319–52. 10.1080/1750984X.2020.1723122

[3] Rudolf K Bickmann P Froböse I Tholl C Wechsler K Grieben C. Demographics and health behavior of video game and eSports players in Germany: the eSports study 2019. Int J Environ Res Public Health. (2020) 17(6):1870. 10.3390/ijerph17061870 32183070 PMC7142975

[4] Trotter MG Coulter TJ Davis PA Poulus DR Polman R. The association between esports participation, health and physical activity behaviour. Int J Environ Res Public Health. (2020) 17(19):7329. 10.3390/ijerph17197329 33049914 PMC7579013

[5] DiFrancisco-Donoghue J Werner WG Douris PC Zwibel H. Esports players, got muscle? Competitive video game players’ physical activity, body fat, bone mineral content, and muscle mass in comparison to matched controls. J Sport Health Sci. (2022) 11(6):725–30. 10.1016/j.jshs.2020.07.006 32711155 PMC9729923

[6] Shulze J Marquez M Ruvalcaba O. The biopsychosocial factors that impact esports players’ well-being: a systematic review. J Glob Sport Manag. (2023) 8(2):478–502. 10.1080/24704067.2021.1991828

[7] Yin K Zi Y Zhuang W Gao Y Tong Y Song L. Linking esports to health risks and benefits: current knowledge and future research needs. J Sport Health Sci. (2020) 9(6):485–8. 10.1016/j.jshs.2020.04.006 32417192 PMC7749245

[8] Poulus DR Sargeant J Zarate D Griffiths MD Stavropoulos V. Burnout profiles among esports players: associations with mental toughness and resilience. J Sports Sci. (2024) 42(18):1685–94. 10.1080/02640414.2024.2405794 39306706

[9] Poulus D Nicholson M Obine E Drew M Fisher K Vella S. Mental health and well-being in esports: a scoping review. Appl Psychol. (2026) 75(1):e70055. 10.1111/apps.70055

[10] Chan G Huo Y Kelly S Leung J Tisdale C Gullo M. The impact of eSports and online video gaming on lifestyle behaviours in youth: a systematic review. Comput Human Behav. (2022) 126:106974. 10.1016/j.chb.2021.106974

[11] Pedraza-Ramirez I Sharpe B Behnke M Toth AJ Poulus DR. The psychology of esports: trends, challenges, and future directions. Psychol Sport Exerc. (2025) 81:102967. 10.1016/j.psychsport.2025.102967 40774402

[12] Leis O Sharpe BT Pelikan V Fritsch J Nicholls AR Poulus D. Stressors and coping strategies in esports: a systematic review. Int Rev Sport Exerc Psychol. (2024) 19:1–31. 10.1080/1750984X.2024.2386528

[13] Poulus DR Sharpe BT Jackman PC Swann C Bennett KJ. Defining elite esports athletes: a scoping review. Int Rev Sport Exerc Psychol. (2024) 19:1–36. 10.1080/1750984X.2024.2386531

[14] WHO Consultation on Obesity, World Health Organization. *Obesity: preventing and managing the global epidemic: report of a WHO consultation (WHO Technical Report Series No. 894)*. WHO Consultation on Obesity; World Health Organization (2000). Available online at: https://iris.who.int/handle/10665/42330 (Accessed June 23, 2026).

[15] Richter A Starker A Schienkiewitz A. Reassessment of alcohol consumption in Germany: which population groups are at increased risk of disease? J Health Monit. (2025) 10(3):e13457. 10.25646/13457 41113342 PMC12530689

[16] Godin G. The Godin-Shephard leisure-time physical activity questionnaire. Health Fit J Can. (2011) 4(1):18–22. 10.14288/hfjc.v4i1.82

[17] Ware JE Jr Snow KK Kosinski M Gandek B. SF-36 Health Survey: Manual and Interpretation Guide. Bosten: The Health Institute, New England Medical Center (1993).

[18] Kroenke K Spitzer RL Williams JB. The PHQ-9: validity of a brief depression severity measure. J Gen Intern Med. (2001) 16(9):606–13. 10.1046/j.1525-1497.2001.016009606.x 11556941 PMC1495268

[19] Buysse DJ Reynolds CF III Monk TH Berman SR Kupfer DJ. The Pittsburgh sleep quality index: a new instrument for psychiatric practice and research. Psychiatry Res. (1989) 28(2):193–213. 10.1016/0165-1781(89)90047-4 2748771

[20] Zimet GD Dahlem NW Zimet SG Farley GK. The multidimensional scale of perceived social support. J Pers Assess. (1988) 52(1):30–41. 10.1207/s15327752jpa5201_2 2280326

[21] Jekauc D Völkle M Lämmle L Woll A. Fehlende Werte in sportwissenschaftlichen Untersuchungen. Sportwissenschaft. (2012) 42:126–36. 10.1007/s12662-012-0249-5

[22] Little RJA. A test of missing completely at random for multivariate data with missing values. J Am Stat Assoc. (1988) 83:1198–202. 10.1080/01621459.1988.10478722

[23] Cohen J. Statistical Power Analysis for the Behavioral Sciences. 2nd edn. Hillsdale: Lawrence Erlbaum Associates (1988).

[24] Keppel G Wickens TD. Design and Analysis: A Researcher’s Handbook. 4th edn. Upper Saddle River: Pearson Prentice Hall (2004).

[25] Nicholson M Thompson C Poulus D Pavey T Robergs R Kelly V. Physical activity and self-determination towards exercise among esports athletes. Sports Med Open. (2024) 10(1):40. 10.1186/s40798-024-00700-0 38625433 PMC11021385

[26] Hidding LM Chinapaw MJ van Poppel MN Mokkink LB Altenburg TM. An updated systematic review of childhood physical activity questionnaires. Sports Med. (2018) 48(12):2797–842. 10.1007/s40279-018-0987-0 30298479 PMC6244567

[27] McNulty C Jenny SE Leis O Poulus D Sondergeld P Nicholson M. Physical exercise and performance in esports players: an initial systematic review. J Electron Gaming Esports. (2023) 1(1):1–11. 10.1123/jege.2022-0014

[28] Lee S Bonnar D Roane B Gradisar M Dunican IC Lastella M. Sleep characteristics and mood of professional esports athletes: a multi-national study. Int J Environ Res Public Health. (2021) 18(2):664. 10.3390/ijerph18020664 33466788 PMC7830734

[29] Kegelaers J Mairesse O Van Ruysevelt L Trotter MG Watson M Pedraza-Ramirez I. The mental health status of esports athletes. J Electron Gaming Esports. (2025) 3(1):1–10. 10.1123/jege.2025-0017

[30] DiFrancisco-Donoghue J Jenny SE Douris PC Ahmad S Yuen K Hassan T. Breaking up prolonged sitting with a 6min walk improves executive function in women and men esports players: a randomised trial. BMJ Open Sport Exerc Med. (2021) 7(3):1–8. 10.1136/bmjsem-2021-001118

[31] Swettenham L Abbott C Leis O. Applied sport psychology in esports. In: Jenny S Besombes N Brock T Cote AC Scholz TM, editors. Routledge Handbook of Esports. London/New York: Routledge (2024). p. 214–24.

[32] Leis O Poulus DR Trotter MG Didymus FF Swettenham LD. Stressors and coping among esports coaches. Psychol Sport Exerc. (2026) 84:103069. 10.1016/j.psychsport.2026.103069 41577061

[33] Bonnar D Lee S Roane BM Blum DJ Kahn M Jang E. Evaluation of a brief sleep intervention designed to improve the sleep, mood, and cognitive performance of esports athletes. Int J Environ Res Public Health. (2022) 19(7):4146. 10.3390/ijerph19074146 35409833 PMC8998799

[34] Swettenham L Whitehead A. Working in esports: developing team cohesion. Case Stud Sport Perform Psychol. (2022) 6(1):36–44. 10.1123/cssep.2021-0023

