# Supplementary material for: Health and health-related behaviors in esports players: an international survey of playtime and nationality differences
Source: Front Sports Act Living. 2026 Jul 8;8:1850349. doi: 10.3389/fspor.2026.1850349 (PMC13387726; doi:10.3389/fspor.2026.1850349)
Supplement: Supplementary file 1 [file Supplementaryfile1.docx]

**Supplement**

*Overview of SF-36 Subscales, Items, and Response Scales*

| Subscale | Items | Response Scale |
| --- | --- | --- |
| Physical Functioning | (1) Does your health now limit you in vigorous activities (e.g., running, lifting heavy objects)? (2) Does your health now limit you in moderate activities (e.g., moving a table, pushing a vacuum cleaner)? | 1 (*strongly disagree*) to 5 (*strongly agree*) |
| Role-Physical | (1) During the past week, did you cut down on the amount of time you spent on work or other activities because of your physical health? (2) During the past week, were you limited in the kind of work or other activities you do because of your physical health? | 1 (*strongly disagree*) to 5 (*strongly agree*) |
| Bodily Pain | (1) How much bodily pain have you had during the past week? (2) How much did pain interfere with your normal work (both outside the home and at home) during the past week? | 1 (*no pain*) to 5 (*very severe pain*) |
| General Health | (1) In general, would you say your health is: (2) Overall, how would you rate your health during the past week? | 1 (*poor*) to 5 (*excellent*) |
| Vitality | (1) How much energy or vitality did you have during the past week? (2) Did you feel worn out during the past week? | (1) 1 (*very low*) to 5 (*very high*) (2) 1 (*never*) to 5 (*always*) |
| Social Functioning | How much did physical health or emotional problems interfere with your social activities (like visiting with friends, relatives)? | 1 (*not at all*) to 5 (*very much*) |
| Mental Health | How much of the time during the past week have you felt calm and peaceful? | 1 (*none of the time*) to 5 (*all of the time*) |

*Note.* All items were adapted from the SF-36 questionnaire (Ware, 1993)
